# Correction: Use of fourth-generation rapid combined antigen and antibody diagnostic tests for the detection of acute HIV infection in a community centre for men who have sex with men, between 2016 and 2019

**DOI:** 10.1371/journal.pone.0258613

**Published:** 2021-10-11

**Authors:** The PLOS ONE Staff

The tenth and eleventh authors, Dante R. Culqui and Joan A. Caylà, should be noted as joint senior authors of this work. The publisher apologizes for the error.
