# Supplementary material for: Use of fourth-generation rapid combined antigen and antibody diagnostic tests for the detection of acute HIV infection in a community centre for men who have sex with men, between 2016 and 2019
Source: PLoS One. 2021 Jul 27;16(7):e0255065. doi: 10.1371/journal.pone.0255065 (PMC8315512; doi:10.1371/journal.pone.0255065)
Supplement: Attachment — Submitted filename: Recenzja PLOS One.pdf [file pone.0255065.s002.pdf]

The reviewed manuscript presents the use of fourth-generation rapid combined antigen and antibody diagnostic tests for the detection of acute HIV infection in a community centre for men who have sex with men. The issue considered is a current and complex problem and is in line with the journal's profile.

1. The content of the article is consistent with the title of the thesis.

2. The summary is correctly edited, it reflects the essence of the article.

3. The purpose of the work has been clearly stated.

4. The issue contained in the article presents the activity of the BCN Checkpoint, which is promoting sexual health among men who have sex with men (MSM) and focuses on the early diagnosis of HIV and the earliest possible initiation of cART. The fourth-generation rapid HIV tests were used for testing, allowing the simultaneous detection of p24 Ag and HIV Ab. This is important for the early detection of HIV infection. The study analyzed three possible groups of positive results and low percentage of people with false positive results. People with confirmed HIV infection were referred to the HIV unit to comence cART on the next working day. The necessity of repeating the test for those who have teted negative was emphasized and attention was drawn to the prompt initiation of cART, which contributes to the improvement of human health and reduces the HIV transmission chain.

5. The conclusions contained in the article are correct and warranted in the content of the thesis.

6. The selection of literature and sources is sufficient and adequate to the problems of paper.

7. The language and style of work are correct.

Manuscript is valuable and well fitted for publication in „PLOS One".
