# Supplementary material for: Use of fourth-generation rapid combined antigen and antibody diagnostic tests for the detection of acute HIV infection in a community centre for men who have sex with men, between 2016 and 2019
Source: PLoS One. 2021 Jul 27;16(7):e0255065. doi: 10.1371/journal.pone.0255065 (PMC8315512; doi:10.1371/journal.pone.0255065)
Supplement: Attachment — Submitted filename: Response to Reviewers.pdf [file pone.0255065.s003.pdf]

Barcelona, 28 April 2021

Thank you for the review and please find below the responses to all issues that were raised:

1. Ensure that your manuscript meets PLOS ONE's style requirements.

Response: Manuscript has been adapted to style requirements.

2. Review your reference list.

Response: Reference list has been checked.

3. Provide an amended Competing Interests Statement.

Response: The updated Competing Interests Statement has been included in the cover letter.

4. List the individual authors and affiliations within this BCN Checkpoint Working Group.

Response: Individual authors and affiliations have been updated.

5. Reviewers' comments:

1. Is the manuscript technically sound, and do the data support the conclusions?

Reviewer #1: Yes
Reviewer #2: Yes

____

2. Has the statistical analysis been performed appropriately and rigorously?

Reviewer #1: Yes
Reviewer #2: Yes

____

3. Have the authors made all data underlying the findings in their manuscript fully available?

Reviewer #1: Yes
Reviewer #2: Yes

____

4. Is the manuscript presented in an intelligible fashion and written in standard English?

Reviewer #1: Yes
Reviewer #2: Yes

____

5. Review Comments to the Author

Reviewer #1: The issue contained in the article presents the activity of the BCN Checkpoint, which is promoting sexual health among men who have sex with men (MSM) and focuses on the early diagnosis of HIV and the earliest possible initiation of cART. The fourth-generation rapid HIV tests were used for testing, allowing the

simultaneous detection of p24 Ag and HIV Ab. This is important for the early detection of HIV infection. The study analyzed three possible groups of positive results and low percentage of people with false positive results. People with confirmed HIV infection were referred to the HIV unit to comence cART on the next working day. The necessity of repeating the test for those who have teted negative was emphasized and attention was drawn to the prompt initiation of cART, which contributes to the improvement of human health and reduces the HIV transmission chain.
The issues contained in the article present the current, especially in the current epidemiological situation. Manuscript is valuable and well fitted for publication in „PLOS One".

Response: Thank you.

Reviewer #2: 1. The study that does identify an important issue which is diagnosing acute HIV infection, include numerous group of participations with data that supports the conclusions.

2.The statistical analysis has been performed appropriately.

3. The authors made all data underlying the findings in their manuscript fully available without restriction.

4. The paper is generally well written and structured. Overall structure of the article is well organized and well balanced.

Comments:

Line 53
Add „combined" to anitiretroviral treatment so that it matches the abbreviation – cART

Response: The word "combined" has been added.

Line 76
Use more formal language

Response: The phrase has been rewritten as requested.

Line 115
Full name should be 'infectious mononucleosis'. Change common name 'flu' to infuenza.

Response: The terms have been changed.

Line 324
There no necessarily to extract gay men from others MSM.

Response: The phrase has been rewritten as requested.

Kind regards,

Jorge Saz Berges
